# Research Trends in the Use of Polyaniline Membrane for Water Treatment Applications: A Scientometric Analysis

**DOI:** 10.3390/membranes12080777

**Published:** 2022-08-12

**Authors:** Muhammad Nihal Naseer, Kingshuk Dutta, Asad A. Zaidi, Muhammad Asif, Ali Alqahtany, Naief A. Aldossary, Rehan Jamil, Saleh H. Alyami, Juhana Jaafar

**Affiliations:** 1 Department of Engineering Sciences, Pakistan Navy Engineering College, National University of Sciences and Technology, Karachi 75300, Pakistan; 2 Advanced Polymer Design and Development Research Laboratory, School for Advanced Research in Petrochemicals, Central Institute of Petrochemicals Engineering and Technology, Bengaluru 562149, Karnataka, India; 3 Department of Mechanical Engineering, Faculty of Engineering Science and Technology, Hamdard University, Madinat al-Hikmah, Karachi 74600, Pakistan; 4 Department of Urban and Regional Planning, College of Architecture and Planning, Imam Abdulrahman Bin Faisal University, P.O. Box 1982, Dammam 31441, Saudi Arabia; 5 Department of Architecture, Faculty of Engineering, Al-Baha University, Al-Baha 65528, Saudi Arabia; 6 Department of Building Engineering, College of Architecture and Planning, Imam Abdulrahman Bin Faisal University, P.O. Box 1982, Dammam 31441, Saudi Arabia; 7 Department of Civil Engineering, College of Engineering, Najran University, Najran 55461, Saudi Arabia; 8 Advanced Membrane Technology Research Centre, School of Chemical and Energy Engineering, Universiti Teknologi Malaysia, Skudai Johor 81310, Malaysia

**Keywords:** polyaniline, membranes, water treatment, bibliometric analysis, bibliographic analysis

## Abstract

Polyaniline (PANI), which is a member of the family of electrically conducting polymers, has been widely discussed as a potential membrane for wastewater treatment. Although a steady growth in PANI literature was observed, analyzing PANI literature quantitatively is still a novelty. The main aim of this study is to unearth the current research status, global trends, and evolution of PANI membranes literature and their use in water treatment applications over time. For this purpose, a scientometric study was performed consisting of bibliometric and bibliographic analysis. A total of 613 entities were extracted from Web of Science published during the last 50 years and were analyzed to map trends based on leading peer-reviewed journals, publication records, leading research disciplines, countries, and organizations. The study shows that the number of annual publications increased exponentially from 2005 to 2020 and is expected to keep increasing in the current decade. The *Journal of Membrane Science* published the highest number of articles and was identified as the most-cited journal in the field. China, India, and the USA were observed as the top three research hubs. The top-ranked authors in the field were Wang, Jixiao, and Wang, Zhi. To find research trends, four different clusters of keywords were generated and analyzed. The top five most frequent keywords turn out to be polyaniline, water, performance, membranes, and nanoparticles. The analysis suggests that the application of nanotechnology for modifying PANI membranes (using nanoparticles, nanotubes, and graphene specifically) is the future of this field. This study elucidates the research streamline of the field that may serve as a quick reference for early career researchers and industries exploring this field.

## 1. Introduction

Ever since its discovery, polyaniline (PANI) has proven to be an exceptionally useful polymer that has found wide applications in diverse fields, such as corrosion-resistant coatings [1], tissue engineering [2], electron conductive catalyst supports [3], proton conductive as well as separation membranes [4,5], sensors [6], etc. The use of PANI as a component of membranes for water treatment application has been widely explored recently, as evident from the discussions in the later sections of this article. In such membranes, PANI functions mainly as a pore regulating entity. In addition, its charged nature and surface wettability help in selectively restricting the passage of certain moieties, while allowing others to pass through easily. In addition, the unique nature of PANI as a material, due to the ability to tune its pore size and surface wettability by varying the electrical field [7], as well as the charge by modulating the pH (i.e., by acid doping/base de-doping) [8], has provided the impetus to employ it in the fabrication of stimuli-responsive membranes for water treatment applications [9]. The use of PANI as a component in water filtration and treatment membranes has been completed either in the form of blends/composites with one or more components (mainly polymers) or as coating/laminates on a base polymeric membrane [4]. When acting as a cation-exchanger, the extent of doping of PANI and the feasibility of the interfacial transfer of charge between PANI and a second component is of extreme importance, and the latter is governed by the area of contact and the interaction between the two phases [10]. In this regard, coating/lamination provides a better scenario as it results in higher surface coverage of PANI, often with high interfacial strength, and the coating/laminating layer of PANI acts as a physical barrier to facilitate restrictive movement of moieties across the membrane [11]. On the other hand, in the case of blend/composite membranes, modulation of the membrane pore size can be better achieved; however, interfacial strength gets often compromised leading to phase separation. In addition, the ability to reversibly modulate the surface wettability and/or charge of PANI by external stimuli, such as electric field and pH, helps in circumventing the fouling issue of PANI-based membranes, and this self-cleaning attribute can be better realized in the case of PANI-coated/laminated membranes [8,12,13]. In effect, polyaniline-based membranes can serve as smart membranes with antifouling and self-cleaning properties, with the exhibition of pore modulation, surface wettability reversal, and charge reversal attributes, resulting in significant flux, flux recovery ability, and satisfactory rate and efficiency of foulant rejection and contaminant removal.

At this juncture, it was felt necessary to sketch an overview of the recent research trends in the use of PANI membranes for water treatment applications. It must be noted here that despite its high value in understanding its research directions, such a study has not been reported so far in the available publications. Keeping this in mind, this article aims at providing quantitative mapping of the field of PANI membranes, in terms of water treatment, by using bibliometric and bibliographic analysis.

Bibliometric analysis is a widely used research tool for exploring and analyzing the literature published on a specific topic or a field to map research evolution while endeavoring emerging topics of a field [14]. This technique of quantitative analysis was introduced in 1969 [15], and since then it has been applied in a wide range of disciplines from cancer [16], and cloud computing [17] to microbial fuel cells [18]. The bibliographic analysis is another important technique that uses the method of data mining and finds out different keywords used in a field. Using this technique, the most frequent keywords can be portrayed quantitatively and their relation to other keywords or important concepts can also be studied. In addition, the timeline of the emergence of these keywords can also be analyzed [18]. Historically, these techniques were limited by the data availability; however, the establishment of databases, such as Web of Science (WoS), has broadened the scope of these techniques [19]. In this study, for data collection, the Core Collection of Clarivate Analytics, and Web of Science were used. WoS is one of the oldest international databases, which is considered a widely acceptable and authoritative source of literature search, citation analysis, and indexing of journals [20,21,22].

## 2. Research Methodology

About 21,100 peer-reviewed journals are currently indexed in this database which provides access to about 53 million publications along with 1.18 billion cited references [18]. The literature related to PANI membranes was searched in 2021-Q4 by using the advanced search option with the following text format:

((((((TS = (aniline black*)) OR TS = (polyaniline*)) OR TS = (PANI*)) OR TS = (poly-aniline*)) AND TS = (membrane*)) AND TS = (water*))

where TS stands for topic search which implies looking for searched terms or schemes in the title, abstract, author keywords, and keywords plus.

**Figure 1:**
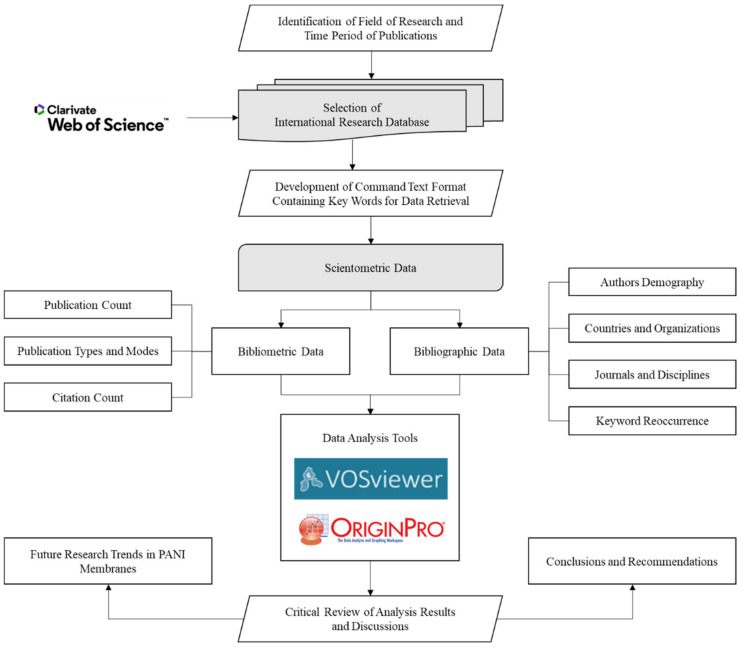
Research methodology followed for the study.

The year of publication was set to 1971–2020, and all the articles published in languages other than English were excluded from the analysis. Data were exported as plain text files: each containing 500 entities. This data included a full record of publication, including the type of article and page numbers along with cited references. For visualizing and analyzing data, VOSviewer [23], and Origin Pro software were used. Mainly, VOSviewer was used for mapping and creating neural networks. In addition, different analyses such as keyword analysis, organization analysis, country analysis, and authorship analysis were also performed using VOSviewer. In all the analyses, redundant terms were excluded. The complete step-by-step procedure followed to perform the research is shown in Figure 1.

## 3. Results and Discussion

On searching the Core Collection of WoS, a total of 613 entities were found related to PANI during the span of 50 years from 1971 to 2020. The scientometric analysis and discussion on the results obtained are being produced as follows.

### 3.1. Publication Count

Although the authors searched the Core Collection of WoS for the last fifty years, the very first article discussing PANI membranes in the context of water was spotted in 1995 [24]. In this article, Schmidt et al. [24] developed a PANI membrane by depositing a homogeneous layer of PANI, formed by polymerization of aniline, on a porous platinum electrode. This pervaporation membrane was tested for the transport of protons and water through it [24]. However, the first article that introduced conducting polymer membranes dates back to 1976 [25]; while, the first patent [26] explaining the method to produce PANI membranes dates back to 1993. In this patent [26], only the methodology for fabricating membranes of PANI was disclosed, and the application of those membranes concerning water treatment was not discussed. In the next year (i.e., 1994), PANI membranes were patented for gas separation [27], but water treatment using PANI remained unpatented till 1998. In this 1998 patent, Kenichi [28], for the first time, discussed that PANI in the form of membrane, powder, or palate can be used for water treatment. In addition, the concept of electrolyzing water using PANI as the cathodic material was also introduced by Kenichi [28].

The last 25 years of research progress in PANI membranes for wastewater treatment can be classified into two periods: the explorative period and the developmental period. The explorative period is the time when the conceptual basis of the topic was being built and the development period is the time when the topic started getting matured and received a lot of attention from the research community. For the present case, 1995–2004 was the explorative period for PANI membranes. On average, one or two new publications were introduced each year during this era. Following the first publication [24] related to PANI, in 1995, as membranes for water treatment, the effect of various parameters such as temperature, doping, and blending on the permeability of PANI membrane was discussed in 1997 [29,30]. It was revealed that doped PANI membranes exhibit better performance in water separation from carboxylic acid as compared to un-doped membranes [29]. The second important concept introduced in 1997 was that blending improves the thermal stability of PANI membranes [30]. These studies [29,30] became the base of almost all studies that were published in the explorative area. In the next 6 years, no new concept was introduced; however, different scientists verified the effect of doping/blending on the performance of PANI membranes. For instance, in 1998, no new concept was introduced; rather, two studies [31,32] were published in which researchers compared the performance of doped and un-doped PANI membranes, and validated the fact that it increases the performance of the membrane. New water supply pipe materials have also been tested for quality [27]. Pervaporation of isopropanol from water [33], ceramic PANI membranes [34], and pervaporation of ethanol from water were discussed in 1999 [35]. Over the next 4 years of the explorative era, the focus was still on comparing doped and un-doped membranes [36,37,38]; however, in 2004, the concept of electrodialysis [38] was coupled with PANI membranes that opened a new research streamline.

**Figure 2:**
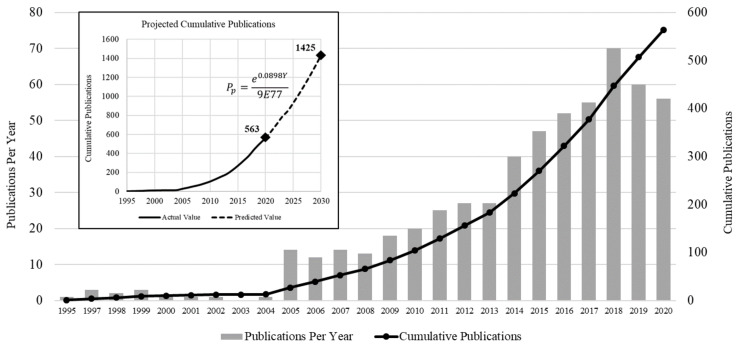
Research growth in PANI membranes for water treatment applications.

The number of articles published annually showing the research growth in the subject under study is plotted on a chart along with the cumulative number of publications starting from 1995 to 2020 as shown in Figure 2.

After performing the statistical analysis of the data of the publications, it is concluded that the cumulative number of publications shows a regular exponential trend with the following equation.

(1) $$P_{p}=\frac{e^{0.0898Y}}{9\times 10^{77}}$$

where *P_p_* is the projected number of cumulative publications in a specific year *Y*. Using Equation (1), the total number of publications that are expected to be published by 2030 is estimated as 1425, which is nearly three times the cumulative number of publications in 2020.

In the development era starting in 2005, steady growth was observed in PANI membrane literature focusing on water treatment. A wide range of studies was performed during this period and various technologies were integrated with it, as discussed in Section 3.7 and Section 4.

### 3.2. Publication Types and Modes

The entities obtained from the database were distributed in six categories: research articles, proceeding papers, review articles, book chapters, meeting abstracts, and early access. The majority of the entities belong to research articles (93.96%), proceeding papers (7.01%), and review articles (2.28%), whereas the contribution of the remaining categories is marginally insignificant, i.e., 0.98%. Only 12.56% of the total entities were published as open access articles, while the remaining 87.43% were accessible with a subscription. Concerning the language of publication, 98.37% were published in the English language, followed by Chinese, Portuguese, and Polish, which contributed 1.14%, 0.32%, and 0.16%, respectively. Articles published in languages other than English were not included in the analysis.

### 3.3. Citation Count

The citation analysis showed that all entities related to PANI were cited 15,059 times with each paper cited by 24.97 times on average. Overall, the h-index of this topic was 60. Most cited articles of a field provide valuable information about the important concepts or ideas of that field. In the case of PANI membranes for water treatment, a list of most cited articles has been presented in Table 1. The most cited article [39] in this field is related to the application of nanocomposites and nanoparticles, along with PANI, for heavy metal removal from wastewater. These key terms were also observed in Section 3.7, denoting frequent keywords used by authors, which implies that the findings are in coordination.

### 3.4. Author Demography

**Figure 3:**
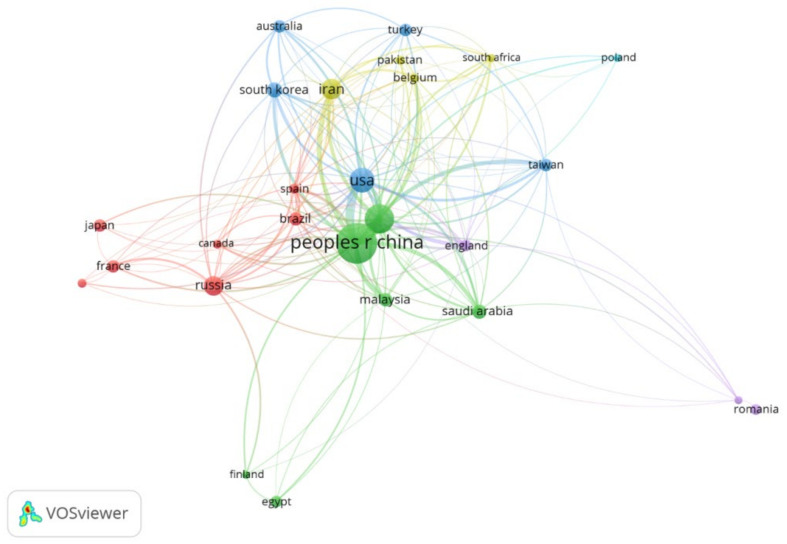
Leading countries and their collaborative network in research on PANI membranes.

**Figure 4:**
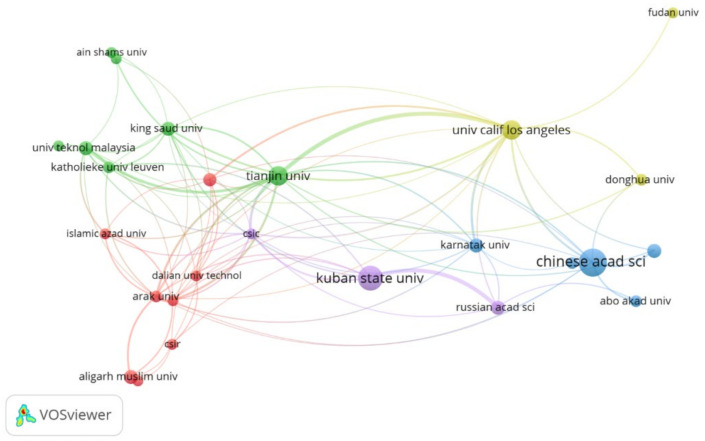
Leading organizations in PANI membranes for water treatment applications.

In author analysis, it was realized that there are about 2551 authors who have contributed to research on PANI membranes for water treatment. A list of the top 10 authors is presented in Table 2. The main research area of the majority of the leading authors is chemical engineering. Out of the top ten authors, four of them belong to China, which is spotted as the leading research country in Figure 3. Moreover, Tianjin University has the highest number of researchers in the list of leading authors in PANI membrane research that validates the findings of Figure 4.

### 3.5. Countries and Organizations

In total, about 63 countries have been involved in PANI research over the last 50 years. To spot out leading countries, only those were selected who have a minimum of seven publications and ten citations on their credit. Twenty-six countries were filtered out and are mapped in Figure 3. The size of the circle denotes the contribution of that country and the line joining two countries shows their collaboration.

It is clear from Figure 3 that there are six main clusters denoting leaders and collaborators in each cluster: cluster 1 is led by Russia with Brazil, Canada, France, Germany, Japan, and Spain as contributors; cluster 2 is led by China with India, Egypt, Finland, Malaysia, and Saudi Arabia as collaborators; cluster 3 is led by the USA with Australia, South Korea, Taiwan, and Turkey as collaborators; cluster 4 is led by Iran with Pakistan, Belgium, South Africa as collaborators; cluster 5 is led by England with Italy and Romania as collaborators, and cluster 6 is led by Poland.

The top 10 countries involved in PANI research contributed 71.39% in publications and 71.37% in citations as shown in Table 3. Overall, China leads the PANI membrane research in terms of both the number of publications and the number of citations. India is the second most contributing country in terms of publications followed by the USA; however, in terms of the number of citations, the USA leads India. Moreover, the normalized contribution was also calculated, which is the ratio of the number of publications to the population. In this analysis, China leads in terms of normalized contribution, followed by Australia, Saudi Arabia, Malaysia, and Iran.

The leading organizations in PANI membrane research are depicted in Figure 4. Overall, the Chinese Academy of Sciences (CAS) holds the leading position, followed by Kuban State University and Tianjin University. It should be noted here that CAS is a group of Chinese research institutes that includes researchers from almost all over China. Tianjin University also comes under the shadow of CAS. A list of the top 10 organizations, ranked based on their contribution, is listed in Table 4.

### 3.6. Journals and Disciplines

**Figure 5:**
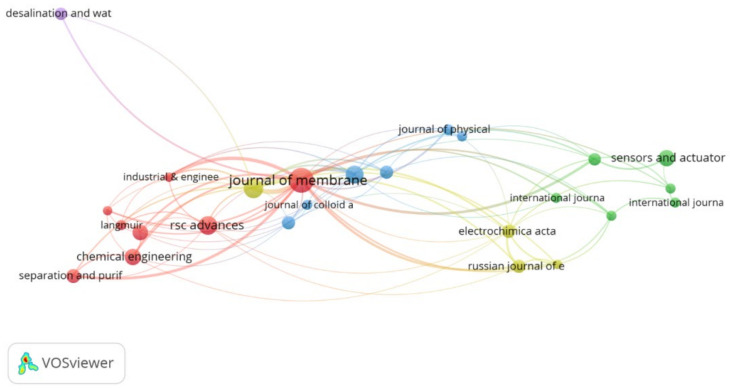
Leading journals in publishing research on PANI membranes for water treatment.

This analysis shows that the literature related to PANI membranes for water treatment was published in a total of 276 journals, out of which 26 have published at least 5 articles and have received at least 50 citations as shown in Figure 5. The top 10 journals contributed 59.25% in documents and 65.42% in citations listed in Table 5. Overall, the *Journal of Membrane Science* is the leading journal in this field, followed by *Desalination* and *RCS Advances*.

Among the top 10 journals in the field, the majority of the journals are focused on the chemistry or chemical aspects of PANI membranes, followed by the materials’ application. Different categories involved in studying the chemical aspects of PANI membranes are electrochemistry, analytical chemistry, physical chemistry, and multidisciplinary chemistry. All these categories cover different aspects of chemistry. For example, electrochemistry deals with the relationship between chemical changes with electricity. Generation of electricity as a result of chemical reactions, and chemical changes as a result of electricity production, are the main topics covered by electrochemistry. Analytical chemistry sheds light on the analytical techniques that generate any type of information about chemical systems, such as separation techniques, pyrolysis, and thermal analysis. Physical chemistry includes resources on photochemistry, solid-state chemistry, kinetics, catalysis, quantum chemistry, surface chemistry, electrochemistry, chemical thermodynamics, thermophysics, colloids, fullerenes, and zeolites. Multidisciplinary chemistry is concerned with the interdisciplinary approaches applied to chemical systems [48].

The engineering perspectives of PANI membranes are discussed under chemical engineering which deals with the conversion of raw materials to useful products. The next research category most popular among the top 10 journals is related to materials including polymer science and multidisciplinary materials science. Polymer science includes all resources dealing with the study, production, and technology of natural or synthetic polymers; whereas, multidisciplinary materials science covers resources having a general or multidisciplinary approach to the study of the nature, behavior, and use of materials [48].

**Figure 6:**
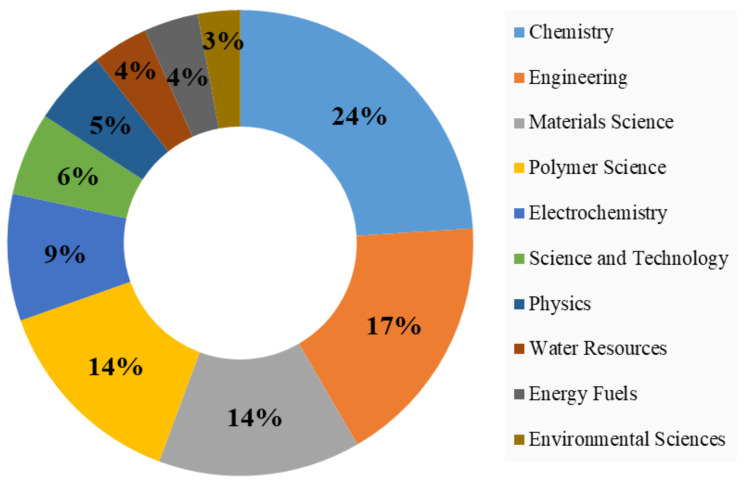
Major research disciplines in PANI membranes for water treatment.

Major research disciplines within PANI research are depicted in Figure 6. Chemistry is the leading discipline, followed by engineering, materials science, and polymer science. The findings of this section reflect the findings listed in Table 5.

### 3.7. Keyword Reoccurrence

Overall, the most frequently used keywords are listed in Table 6, and these keywords contribute about 57.33% of all keywords. The analysis shows that the main concern of the research community has been to improve the performance of PANI membranes and to introduce improved fabrication processes. The applications of nanoparticles and nanocomposites are also prominent.

**Figure 7:**
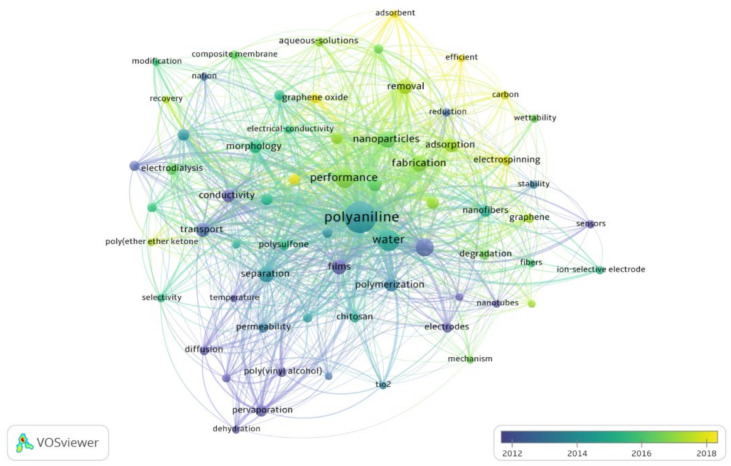
Research evaluation in PANI membranes for water treatment applications.

In addition, information related to the evolution of the field can also be extracted from content analysis as shown in Figure 7. In the early years of this decade, the main focus of researchers was to develop membranes by electro-polymerization and to study the effect of temperature, diffusion, and blending of membranes. The most famous technique of this era was dehydration and pervaporation. Following this initial phase, the attention slowly shifted towards performance optimization; as a result, nanoparticles and carbon nanotubes were introduced. In the last few years of this decade, desalination captured the attention. Other hot topics of this time include applications of graphene oxide and electrospinning, and the introduction of new adsorbents.

## 4. Future Research Trends of PANI Membranes in Water Treatment

**Figure 8:**
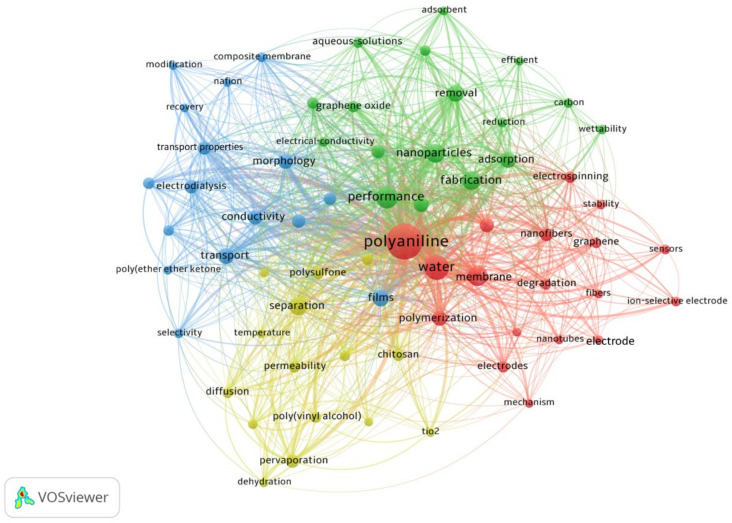
Most frequently used keywords in PANI membranes for water treatment applications.

The content analysis technique was used to find out the main research streams of the field. A total of 3215 keywords were extracted from the analysis and a filter was applied to map frequent keywords. Only those keywords sorted out have a minimum frequency of occurrence of 10, and these are presented in Figure 8. The radius of the circle shows the frequency of occurrence, while the line joining two circles shows the co-occurrence of keywords. A larger circle or thicker line implies a higher frequency of occurrence. There are four different colors used in Figure 8, each denoting a cluster.

Cluster 1 (red) relates carbon nanotubes to PANI membranes and water treatment. A strong linkage was observed between PANI membranes and carbon nanotubes reflecting the fact that it is a common practice in the literature to reinforce, template, or blend carbon nanotubes with PANI [49,50,51,52]. In blending, the amount of nanofiber is considered to be a crucial factor because it determines the characteristics of the membrane, such as thermal stability, chemical structure, hydrophilicity, and porosity [49]. Therefore, carbon nanotubes are mapped to nanofibers as shown in Figure 6. Moreover, cluster 1 highlights the fact that polymerization, electrospinning, and electro-polymerization are among some famous methods used in the preparation, synthesis, and characterization of PANI membranes. Another important piece of information extracted from cluster 1 is that the application of graphene has been promising in this field, with its strong correlation with the performance, stability, and degradation of PANI membranes. Studies show that graphene-based PANI membranes exhibit improved structure and electrochemical performance [53,54,55,56].

Cluster 2 (green) portrays methods used for the removal or reduction of pollutants from wastewater using PANI membranes. Mostly discussed methods/techniques in this regard are adsorption, ultrafiltration, and nanofiltration. In addition, cluster 2 also highlights that electrical conductivity and wettability play vital roles in determining water treatment efficiency. Cluster 3 (blue) sheds light on the different techniques used to improve the efficiency or performance of PANI membranes. The study of morphological characteristics, surface modification, and transport properties of PANI membranes has been promising and mostly discussed by the research community. Cluster 4 (yellow) also highlights some common practices used in coordination with PANI membranes for water purification. Dehydration and pervaporation of water-miscible compounds such as tetrahydrofuran, as well as separation and pervaporation of gasses and liquids, are found to be some most prominent practices. Moreover, hydrophilicity retention of PANI membranes due to chitosan, along with the effect of temperature and permeability of membranes are spotted as some highly discussed topics.

## 5. Key Challenges and Way Forward

PANI membranes have great potential as an alternative to traditional oxidants for oxidation of organic compounds in wastewater treatment. PANI membranes can be applied at higher pH values than other available oxidants, and it also has advantages such as fast redox reaction kinetics and low cost. These advantages have led to the use of PANI-based technologies for removal of organic compounds from aqueous solutions. The main applications are in dyeing, food and beverage, pulp and paper, textile, chemical, petrochemical, and pharmaceutical industries. However, there are a few challenges to using PANI membranes for the treatment of wastewaters as discussed below.

Despite the great potential of PANI membranes, the use of PANI membranes for wastewater treatment is limited due to the challenges associated with their application. The main challenge is the reduction of the lifetime of the membrane by the oxidation of the PANI. The use of antioxidants is another challenge, and the feed solution may be limited in the number of antioxidants available. The PANI is a semiconductor and is sensitive to electromagnetic fields (EMFs). This makes the PANI membranes prone to breakdown when the membranes are subjected to EMFs. Although the PANI membranes have great potential, their application in wastewater treatment is still in its infancy. Some key challenges of using PANI membranes for the treatment of wastewaters are discussed below.

### 5.1. Poor Durability and Stability of PANI Membranes

The main factors affecting the PANI membrane durability are the chemical nature of the species transported, the pH of the solution, and the redox potential of the membrane. These factors lead to PANI degradation, which includes loss of electrochemical activity followed by a decrease in the crosslinking density and PANI content in the membrane. The main mechanisms of PANI degradation are reduction in functional groups, oxidation of the aromatic rings, and possible chain scission. The reduction in functional groups by water-soluble species can be prevented by keeping the pH low. Oxidation of the aromatic rings can be prevented by adding antioxidants such as phenol, hydroquinone, and thiabendazole. Further, it can also be controlled by reducing the pH of the solution. Lastly, PANI chain scission can be controlled by low pH values, but is a very slow process. The chain scission leads to the formation of highly branched PANI polymers and poor water permeability. When these branched polymers react with other oxidants, the membrane loses its ability to remove organic impurities.

Owing to this degradation, the life span and durability of PANI membranes become a challenge. Therefore, there is a dire need to develop new functional groups and aromatic rings that could help in increasing PANI membrane stability.

### 5.2. Lack of Reliable Techniques to Determine PANI Concentration

Due to the high pH and conductivity of the feed solution, the selection of an appropriate electrochemical technique to determine PANI concentration becomes a challenge.

The PANI content in the membrane can be quantified by UV–Vis spectroscopy for the UV–Vis region of the spectrum. However, this technique does not provide information about the amount of PANI degraded in the membrane. To overcome these limitations, Fourier transform infrared spectroscopy (FTIR) and X-ray photoelectron spectroscopy (XPS) have been used to some extent. However, these techniques are time-consuming and expensive. In addition, they do not provide precise information about PANI degradation.

### 5.3. PANI Degradation and Regeneration

The oxidation of the PANI in the membrane leads to the formation of PANI radicals, which react with water to form hydroxyl radicals which reduce the lifetime of the membrane.

The regeneration of the PANI membranes is an important issue. The main regeneration methods are air oxidation, UV oxidation, and chemical oxidation. Air oxidation is a viable method to regenerate the PANI membranes. However, it is not advisable for wastewater treatment applications due to the high pH value of the feed solution. UV oxidation is the common method used for the regeneration of the PANI membranes. It is a simple method, but it requires a high UV intensity and is not suitable for large-scale applications. Low-pressure chemical oxidation is a promising method for the regeneration of PANI membranes. This method is based on the oxidation of phenol by ozone in water to generate HO radicals, which react with the PANI to form phenol and regenerate the PANI. However, requiring low pressure is a key limitation of this process.

### 5.4. Adverse Environmental Conditions in Wastewater Treatment

For industrial applications, PANI membranes should have the potential to deal with wastewater of diverse contamination levels and conditions. The adverse environmental conditions found in wastewater treatment are biological activity, high pH values, temperature, and pressure. The biological activity can be controlled by using antibiotics and biocides, which are not suitable to be used with PANI membranes. The pH of the feed solution is difficult to be controlled due to the chemical reactions and microbial activity. The temperature and pressure can be controlled by using jacketed reactors, but they are also challenging factors.

Further, PANI membranes have limitations when the feed water is highly saline and has high pH values. In the presence of seawater in the feed solution, the membrane is less stable due to the presence of NaCl, which can interact with the PANI in the membrane and form NaPANI. The high pH feed solution leads to the formation of hydroxide ions, which can react with the PANI and form water-soluble PANI species with anions such as chloride. This results in the formation of pores in the membrane and membrane fouling.

## 6. Conclusions

This study reports a scientometric analysis of the literature, related to the application of PANI membranes for water treatment and published during the last 50 years. A total of 613 entities were extracted from the Web of Science and were analyzed using bibliometric and bibliographic analysis techniques. The key findings of this study are as follows:

- The research growth in the application of PANI membranes for water treatment is highlighted. The analysis reveals that the concept of using PANI membranes for water treatment application was introduced during the 1990s. The era of 1995–2005 was an explorative period in which conceptual basics were developed, such as PANI membrane doping and blending, whereas, after 2005, the development era started in which this topic received steady attention.
- A total of 276 journals have published research related to this topic out of which *Journal of Membrane Sciences, Desalination,* and *RSC Advances* are the leading journals. The majority of the top journals in this field were focused on chemical aspects of membranes followed by material applications in PANI membranes.
- A total of 63 countries were found active in PANI membrane research with China, India, and the USA as top players in terms of citations and number of publications. Nevertheless, concerning the number of publications per population, China and Australia were spotted as the leaders.
- Concerning research trends, performance optimization of PANI membranes for water treatment was observed as the most discussed topic in the literature. For performance enhancement, the immensely used techniques include the use of nanoparticles and nanocomposites, modification in fabrication techniques, and the use of films.
- Using nanoparticles, carbon nanotubes, and graphene oxides; employing electrospinning, and introducing new adsorbents are hot topics in the field.

## Figures and Tables

**Table 1:** Most cited articles related to PANI membranes.

Rank	Title	Citations	Avg. Citation per Year	Ref.
1	Novel polyethersulfone nanocomposite membrane prepared by PANI/Fe_3_O_4_ nanoparticles with enhanced performance for Cu(II) removal from water	202	20.2	[39]
2	Performance improvement of polysulfone ultrafiltration membrane by blending with polyaniline nanofibers	158	11.3	[40]
3	Sulfonated poly (ether ether ketone)/polyaniline composite proton-exchange membrane	134	8.38	[41]
4	Novel dense poly (vinyl alcohol)-TiO_2_ mixed matrix membranes for pervaporation separation of water-isopropanol mixtures at 30 °C	122	7.63	[42]
5	Ions and solvent transport across conducting polymers investigated by AC electrogravimetry. Application to polyaniline	108	4.91	[43]
6	Pore-structure, hydrophilicity, and particle filtration characteristics of polyaniline-polysulfone ultrafiltration membranes	86	7.17	[44]
7	Pervaporation separation of water plus isopropanol mixtures using novel nanocomposite membranes of poly (vinyl alcohol) and polyaniline	84	4.94	[45]
8	Removal of aqueous Hg(II) and Cr(VI) using phytic acid doped polyaniline/cellulose acetate composite membrane	83	10.4	[46]
9	Hierarchical Composite Polyaniline-(Electrospun Polystyrene) Fibers Applied to Heavy Metal Remediation	81	11.6	[47]
10	Electric Field Induced Switchable Wettability to Water on the Polyaniline Membrane and Oil/Water Separation	79	13.2	[7]

**Table 2:** Leading authors of research on PANI membranes for water treatment.

Name	Organization	Research Area
Wang, Jixiao	Tianjin University	Chemical engineering, polymers
Wang, Zhi	Tianjin University	Chemical engineering
Kononenko, Natalia A	Kuban State University	Physical chemistry
Zhao, Song	Tianjin University	Modeling and simulation
Wang, Shi-Chang	Tianjin University	Chemical engineering, membrane science
Huang, Shin-Chin	National University Kaohsiung	Chemical engineering, membrane science
Kaner, Rb	University of California LA	Material science
Ball, I. Joseph	University of California LA	Chemical engineering, material science
Berezina, Ninel P.	Kuban State University	Physical chemistry
Van Der Bruggen, Bart	Tshwane University of Technology	Chemical engineering

**Table 3:** Leading countries in research on PANI membranes for water treatment.

Rank	Country	Publication Contribution	Citation Contribution	Normalized Contribution
1	China	23.41%	22.98%	1.28 × 10^−7^
2	India	12.74%	11.91%	7.1 × 10^−8^
3	USA	9.10%	13.23%	2.12 × 10^−7^
4	Iran	6.11%	5.62%	5.6 × 10^−7^
5	Russia	5.59%	3.08%	2.95 × 10^−7^
6	South Korea	3.51%	3.12%	5.21 × 10^−7^
7	Saudi Arabia	3.12%	1.46%	6.89 × 10^−7^
8	Brazil	2.73%	4.29%	9.88 × 10^−8^
9	Malaysia	2.73%	2.86%	6.49 × 10^−7^
10	Australia	2.34%	2.83%	7.01 × 10^−7^

**Table 4:** Leading organizations in research on PANI membranes for water treatment applications.

Rank	Organization	Country	Publication Contribution	Citation Contribution
1	Chinese Academy of Sciences	China	8.51%	10.18%
2	Kuban State University	Russia	7.09%	3.64%
3	Tianjin University	China	4.96%	9.72%
4	University of California LA	USA	4.96%	6.66%
5	Politechnica University of Bucharest	Romania	4.26%	1.53%
6	Aligarh Muslim University	India	3.19%	3.89%
7	Karnatak University	India	3.19%	7.61%
8	King Saud University	Saudi Arabia	3.19%	1.43%
9	Russian Academy of Sciences	Russia	3.19%	2.02%
10	Universiti Teknologi Malaysia	Malaysia	3.19%	2.58%

**Table 5:** Leading journals in publishing research on PANI membranes for water treatment.

Rank	Journal Name	Impact Factor	Document Contribution	Citation Contribution	Category
1	*Journal of Membrane Science*	8.74	11.85%	21.91%	Polymer science Engineering, chemical
2	*Desalination*	9.5	7.41%	7.01%	Water resources Engineering, chemical
3	*RSC Advances*	3.36	7.04%	5.24%	Chemistry, multidisciplinary
4	*Journal of Applied Polymer Science*	3.13	6.30%	3.43%	Polymer science
5	*Chemical Engineering Journal*	13.3	5.19%	3.41%	Engineering, environmental engineering, chemical
6	*Sensors and Actuators B-Chemical*	7.46	5.19%	7.21%	Chemistry, analytical electrochemistry instruments and instrumentation
7	*ACS Applied Materials & Interfaces*	9.23	4.81%	7.26%	Materials science, multidisciplinary nanoscience and nanotechnology
8	*Separation and Purification Technology*	7.31	4.07%	3.74%	Engineering, chemical
9	*Electrochimica Acta*	6.9	3.70%	2.62%	Electrochemistry
10	*Journal of Materials Chemistry A*	12.7	3.70%	3.60%	Materials science, multidisciplinary chemistry, physical Energy and fuels

**Table 6:** Most frequent keywords used in PANI for water treatment research.

Rank	Keywords	% Age Occurrence
1	Polyaniline	16.73
2	Water	6.52
3	Performance	4.79
4	Membranes	4.00
5	Nanoparticles	3.10
6	Fabrication	3.05
7	Separation	2.68
8	Films	2.63
9	Removal	2.37
10	Adsorption	2.31
11	Transport properties	2.10
12	Polymerization	1.84
13	Morphology	1.79
14	Nanocomposite	1.79
15	Conductivity	1.63

## Data Availability

The datasets generated and analyzed during the research are available with the corresponding author and can be furnished upon request.
